# Soft agar colony formation assay to quantify mouse embryonic fibroblast transformation after *Salmonella* infection

**DOI:** 10.1016/j.xpro.2023.102379

**Published:** 2023-06-26

**Authors:** Virginie Stévenin, Jacques Neefjes

**Affiliations:** 1 Department of Cell and Chemical Biology, Oncode Institute, Leiden University Medical Center (LUMC), Leiden 2333ZC, the Netherlands

**Keywords:** Cell Culture, Cancer, Microbiology

## Abstract

Links between bacterial infections and cancer are actively investigated. Cost-effective assays to quantify bacterial oncogenic potential can shed new light on these links. Here, we present a soft agar colony formation assay to quantify mouse embryonic fibroblast transformation after *Salmonella* Typhimurium infection. We describe how to infect and seed cells in soft agar for anchorage-independent growth, a hallmark of cell transformation. We further detail automated cell colony enumeration. This protocol is adaptable to other bacteria or host cells.

For complete details on the use and execution of this protocol, please refer to Van Elsland et al.^1^

## Highlights

• Infection of mouse embryonic fibroblasts (MEF) with *Salmonella* Typhimurium
• Seeding MEF post-infection in soft agar for anchorage-independent growth assay
• Automated acquisition and counting of MEF colonies formed in soft agar
• Cost-effective assessment of cell transformation upon bacterial infection

## Abstract (editor highlights)

Publisher’s note: Undertaking any experimental protocol requires adherence to local institutional guidelines for laboratory safety and ethics.

## Graphical abstract

**Figure:**
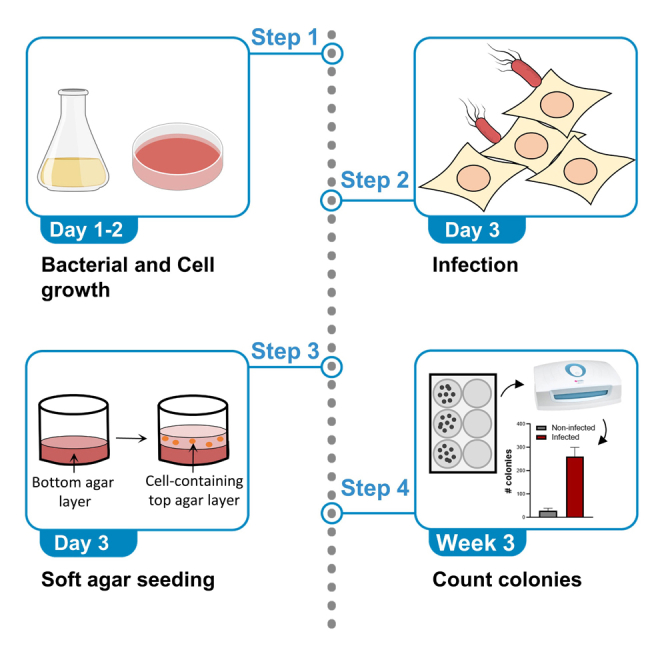

## Before you begin

The protocol below describes the specific steps for quantifying Mouse Embryonic Fibroblast (MEF) transformation upon *Salmonella enterica* serovar Typhimurium infection.^1^^,^^2^ However, we have also used this protocol with multiple *Salmonella enterica* serovars (unpublished).

Here, the described protocol compares 2 conditions: infected and non-infected cells. However, this protocol can be used to compare additional conditions such as different multiplicity of infection (MOI), bacterial strains, inhibitor treatments, etc. In case additional conditions are included, the number of wells and volumes provided should be adapted proportionally. The format used is a 12-well plate for cell infection and a 6-well plate for MEF colony formation in soft agar. This format is ideal to detect a sufficient number of cell colonies (circa 10^2^ per well) in our system (i.e., *Salmonella* infection of fibroblasts). In case of a more efficient transformation, we recommend lowering the number of cells seeded in soft agar by a factor of 2–10 and keeping the 6-well plate format for high reproducibility. A non-infected condition should always be included as a negative control.

Before you begin, determine the number of conditions to compare, prepare solutions, and set up cell and bacterial cultures.

The protocol provides recommended MOI for the infection. However, users should establish the ideal MOI depending on the bacteria infection efficiency and the cell permissivity before the execution of the protocol. This can be done by performing a gentamicin protection assay.^3^

### Cell culture

**Timing: 30 min, 2 days before the experiment.**

**CRITICAL:** Cells should be in culture for at least 10 days (i.e., 2 passages) before performing the experiment. Cells should not experience overcrowding or starvation before the experiment. Cell passages should remain low (below 20) to maximize reproducibility.

1. Maintain cells in culture in DMEM + 10% FBS at 37°C, 5% CO_2_. Split cells regularly and at 80% confluence.

***Note:*** Some cell lines can also withstand 8% FBS instead of 10% with minimal impact on cell behavior allowing to reduce cell culture cost. This can be tested before performing the protocol.

2. 2 days before the experiment, trypsinize and count the cells.
3. Plate 5 × 10^4^ cells per well of a 12-well plate, and complete the volume to 1.5 mL per well. Use 2 wells per condition that will be compared (here, 4 wells).
4. Keep the plate at 37°C, 5% CO_2_ for 2 days, until the experiment.

### Bacterial culture

**Timing: 5 min, 2 days before the experiment; and 5 min, 1 day before the experiment.**

**CRITICAL:***S.* Typhimurium is a human pathogen and should be handled under a sterile laminar BSL-II hood.

**CRITICAL:** To avoid cross-contamination of the *S*. Typhimurium culture, all steps should be performed with sterile material. This is particularly critical if no antibiotics are used for selection.

***Optional:*** In case the *S*. Typhimurium strain contains an antibiotic resistance cassette (for instance in a plasmid coding for a fluorophore), the LB medium and LB-agar should be supplemented with the corresponding antibiotic.

5. At least 2 days before the experiment, put the bacteria in culture.
  a. Starting from a vial of glycerol stock of *S.* Typhimurium stored at −80°C, streak the bacteria on an LB-agar plate containing the appropriate antibiotic (if any) to form individual bacterial colonies.
  b. Incubate the plate at 37°C overnight.
  c. The next day, store the plate at 4°C for up to 1 week.
6. The evening before the experiment, prepare the overnight bacterial culture.
  a. Put 5 mL of LB supplemented with the appropriate antibiotic (if any) in a 50 mL centrifuge tube.
    ***Note:*** Using a 50 mL centrifuge tube increases the air-liquid interphase and will significantly impact the bacteria's speed of growth.
  b. Pick 2–3 bacterial colonies from the LB-agar plate using the same inoculation loop and add them to the LB-containing 50 mL centrifuge tube.
  c. Put the unscrewed cap on the centrifuge tube and attach it with tape to allow air exchange.
  d. Incubate the LB-containing centrifuge tube at 37°C under agitation (220 rpm) overnight (typically 14–20 h).

## Key resources table

**Table:** 

REAGENT or RESOURCE	SOURCE	IDENTIFIER
**Bacterial and virus strains**		

*Salmonella enterica* serovar Typhimurium SL1344	ATCC	ATCC SL1344

**Chemicals, peptides, and recombinant proteins**		

Phosphate-buffered saline (PBS)	Thermo Fisher Scientific	Cat#70011044
Dulbecco’s Modified Eagle’s Medium (DMEM), High Glucose, GlutaMax Supplement	Thermo Fisher Scientific	Cat#10566016
Fetal bovine serum	Sigma-Aldrich	Cat#F7524
0.05% Trypsin-EDTA	Thermo Fisher Scientific	Cat#25300054
Gentamicin	Gibco	Cat#15710064
Ultrapure low-melting-point agarose	UltraPure™, Invitrogen	Cat#16520050
Tryptone	BD	Cat#211705
NaCl	Sigma-Aldrich	Cat#746398
Yeast extract	BD	Cat#212750
Bacto agar	BD	Cat#214010
Primocin	InvivoGen	Cat#ant-pm-05

**Experimental models: Cell lines**		

Mouse embryonic fibroblasts (MEFs) Arf−/−; cMyc+ (recommended passage range 2–20)	Neefjes Lab Scanu et al.^2^	N/A

**Software and algorithms**		

GelCount Software	Oxford Optronix	https://www.oxford-optronix.com/gelcount-cell-colony-counter
GraphPad Prism 5	GraphPad Software	https://www.graphpad.com

**Other**		

Bio-safety cabinet	Thermo Fisher Scientific	1300 Series Class II Type A2
Incubator set at 37°C, 5% CO_2_	Thermo Fisher Scientific	Cat#NC0689918
Centrifuge 5810/5810 R	Eppendorf	5810/ 5810 R
Incubator with an orbital shaker	INFORS, Multitron	112569-3
Photometer	Eppendorf	AG 22331
Centrifuge 5424/ 5424 R	Eppendorf	5424/5424 R
GelCount: Mammalian-cell colony, spheroid and organoid counter	Oxford Optronix	https://www.oxford-optronix.com/gelcount-cell-colony-counter
Water bath at 37°C	N/A	N/A
Water bath at 42°C (optional)	N/A	N/A
Thermomixer compact equipped with Eppendorf SmartBlock 50 mL (optional)	Eppendorf	ThermoMixer® C, Cat#5365000028
Cell density meter	Biochrom	Ultrospec 10
Inoculating loops	SARSTEDT	Cat#86.1562.010
Round Petri plate	Corning, Gosselin™	Cat#BP93B-102
12-well tissue culture treated plate with lid, clear, sterile	Greiner Bio-One	Cat#665180
6-well clear flat bottom not treated cell multiwell culture plate, with lid, sterile	Falcon	Cat#351146
5 mL polystyrene round-bottom tube with cell-strainer cap	Corning, Falcon	Cat#352235

## Materials and equipment

***Note:*** The majority of the equipment described in the key resources table can be replaced by equivalent equipment from other brands. However, the protocol describes specifically the use of the GelCount machine and software for cell colony detection and quantification.

• **DMEM- and DMEM+FBS**: transfer 50 mL of DMEM from a 500 mL bottle to a 50 mL centrifuge tube labeled “DMEM-“. Supplement the remaining 450 mL DMEM with 50 mL of Fetal Bovine Serum (FBS) and label the bottle “DMEM+FBS”.

Storage conditions: 4°C, 1 month.

• **DMEM+FBS+Gent100**: transfer 5 mL of DMEM+FBS in a 15 mL centrifuge tube labeled “DMEM+FBS+Gent100” and supplement with 5 μL of Gentamicin at 10 mg/mL for a final concentration of 100 μg/mL. Vortex.

Storage conditions: prepare fresh on the day of the infection.

***Note:*** The volume here is calculated for 2 conditions. If more conditions are compared, increase the volume proportionally.

• **DMEM+FBS+Gent10**: transfer 50 mL of DMEM+FBS in a 50 mL centrifuge tube labeled “DMEM+FBS+Gent10” and supplement with 5 μL of Gentamicin at 10 mg/mL for a final concentration of 10 μg/mL. Vortex.
Storage conditions: prepare fresh on the day of the infection.

***Note:*** The volume here is calculated for 2 conditions. If more conditions are compared, increase the volume proportionally.

**CRITICAL:** The cell culture medium (DMEM) should not contain antibiotics to avoid interference with the infection steps.

***Note:*** Usually, LB medium and LB-agar plates are prepared in-house by research facilities. Below are common guidelines for LB medium and LB-agar plate preparation.

• Lennox Broth (LB) medium

**Table:** 

Reagent	Final concentration	Amount
Tryptone	1%	10 g
Yeast Extract	0.5%	5 g
NaCl	0.5%	5 g
ddH_2_O	N/A	qsp 1 L
**Total**	**N/A**	**1 L**

Adjust the pH to 7.4, Autoclave for 20 min at 120°C.

Storage conditions: Store at 4°C for a few months.

• LB-agar

**Table:** 

Reagent	Final concentration	Amount
Tryptone	1%	10 g
Yeast Extract	0.5%	5 g
NaCl	0.5%	5 g
Bacto Agar	1.5%	15 g
ddH_2_O	N/A	qsp 1 L
**Total**	**N/A**	**1 L**

Adjust the pH to 7.4, Autoclave for 20 min at 120°C.

Storage conditions: Store at 4°C for a few months.

• To prepare **LB-agar plates**, melt the LB-agar in a double boiler for 13 min in the microwave at 700 W.
• Wait until the medium cools down (circa 30 min at room temperature (RT)).
  ***Optional:*** add antibiotics at the desired concentration and swirl the LB-agar solution to ensure the antibiotic is well mixed.
• Pour agar into round Petri plates.
• Allow plates to sit overnight at RT.

Storage conditions: store at 4°C for 1–2 months.

***Note:*** In this protocol, RT refers to a temperature range of 18°C–22°C.

• To prepare a **3% agar solution**, weight 3 g of ultrapure low melting point agarose powder.
• Transfer to a 250 mL glass bottle.
• Add 100 mL ddH_2_O.
• Autoclave using a liquid program; unscrew the bottle cap and use autoclave tape.
• Let the solution cool down at RT.

Storage conditions: store at RT for 1–2 months.

***Note:*** The agar powder will not dissolve at RT but will dissolve during autoclaving.

The solution can be reused a couple of times, but in time the concentration of agar will increase due to evaporation during heating. Then, a new 3% agar solution should be made.

## Step-by-step method details

### Bacterial subculture

**Timing: 5 min, start 3 h before infection time.**

1. Subculture the bacteria until it reaches an optical density (OD600) of 2, optimal for cell infection.^4^ Troubleshooting 1.
  a. Dilute 150 μL of the overnight culture in 5 mL of LB in a 50 mL centrifuge tube supplemented with the adequate antibiotic, if any.
  b. Grow the bacteria for 3 h in an incubator with an orbital shaker at 37°C, 220 rpm.

### Agar bottom layer

**Timing: 30 min**

The soft agar experiment will consist of 2 layers of soft agar. A 0.7% bottom layer, which will prevent the cells from sinking to the bottom of the well, and a top layer of 0.45%, which contains the cells. The bottom layer should be prepared during the bacterial subculture time.

2. Warm up solutions to reach a stable temperature
  a. Pre-heat growth medium (DMEM-; DMEM+FBS+Gent100 and DMEM+FBS+Gent10) to 37°C.
  b. Melt 3% soft agar solution using a microwave. Swirl the solution every 10–20 s to avoid clumps (Figures 1A–1C). Avoid boiling.

**Figure 1:**
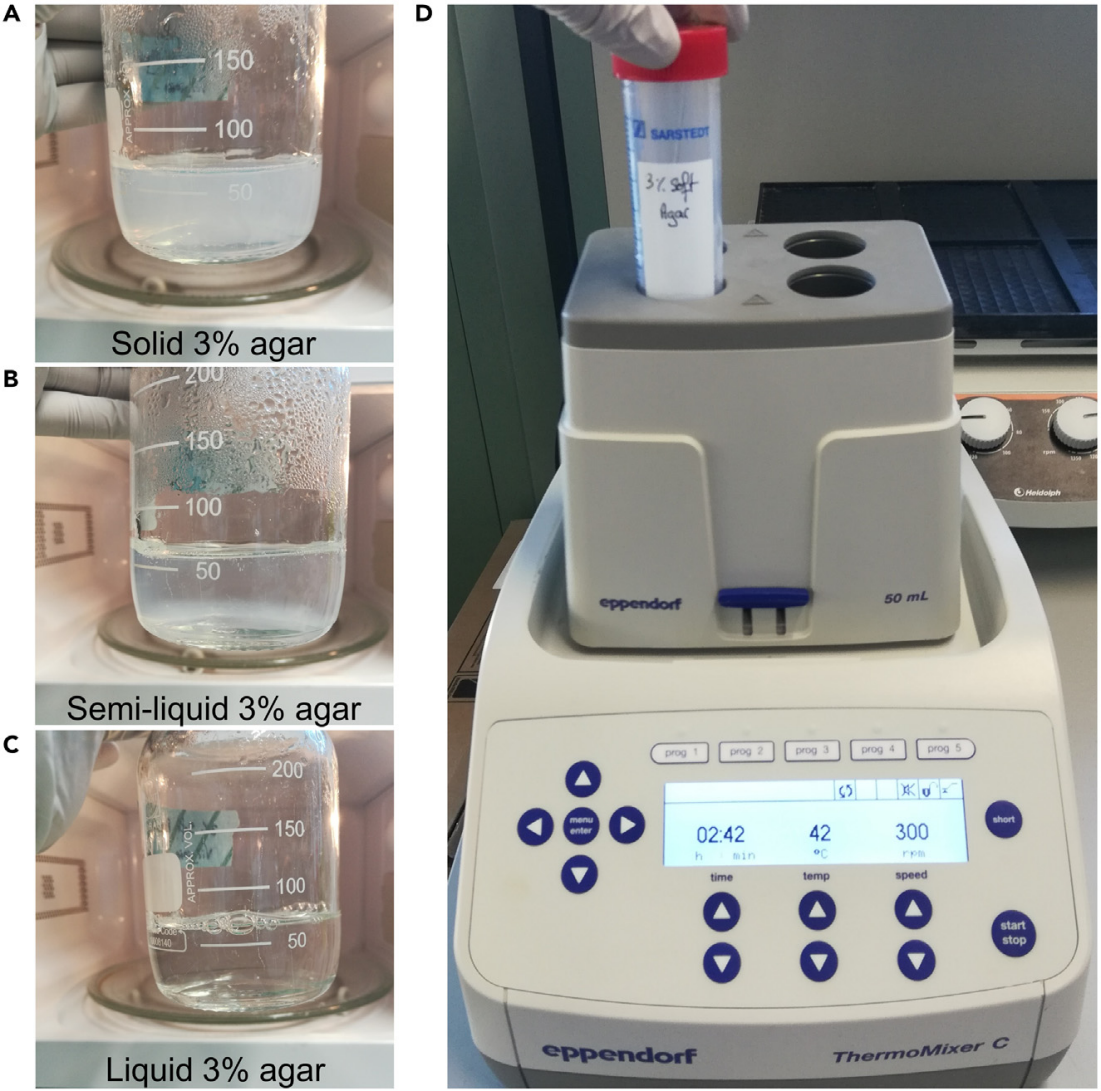
Melting the 3% soft agar solution (A) 3% soft agar solution before melting. (B) 3% soft agar solution checked during melting. (C) 3% soft agar solution fully melted. (D) ThermoMixer used to maintain the 3% soft agar solution at 42°C. Related to Step 2.
  c. Let the 3% soft agar solution cool down using a 42°C water bath or a ThermoMixer with 300 rpm agitation (Figure 1D).
3. Prepare the 0.7% agar bottom layer by diluting 5 mL of the 3% soft agar solution in 16.5 mL of DMEM+FBS+Gent10. Mix gently by pipetting up and down until the solution is homogeneous. Avoid bubbles as much as possible. Do not invert the tube. Act fast as the solution will start solidifying within a few min at RT.
4. Directly pour 3 mL of the mix into each well of a 6-well plate. The bottom of each well should be completely covered.

**CRITICAL:** Avoid air bubbles by gently pouring the solution by applying the tip of the pipette to the wall of the well. Fill the pipette with 1 extra mL to avoid completely emptying the pipette when filling the well. Troubleshooting 2

***Note:*** Use 3 wells per condition. Here 2 conditions (infected and non-infected cells) are compared so a complete 6-well plate is used.

5. Carefully transfer the 6-well plate to a flat shelf of a fridge at 4°C.
6. Let the plate cool down for 20 min until the bottom layer is fully solidified. Troubleshooting 3.
7. Transfer the plate into the incubator at 37°C, 5% CO_2_, with internal humidity of circa 90%.

***Note:*** Keeping similar timing for bacterial growth will increase experimental reproducibility.

### Bacterial infection

**Timing: 4 h**

8. At the end of the 3 h bacterial subculture, measure the concentration of bacteria.
  a. Fill a cuvette with 1 mL LB medium.
  b. Blank the spectrophotometer.
  c. Fill a cuvette with 200 μL of the bacterial subculture in 800 μL of LB medium (volume final 1000 μL, dilution 1:5).
  d. Seal the cuvette with parafilm and measure the OD600. The 1:5 dilution should have an OD600 of circa 0.4, corresponding to the late log phase of the bacterial growth. Troubleshooting 1.
9. Transfer 1 mL of the bacterial subculture to a 1.5 mL microcentrifuge tube.
10. Spin down for 1 min at 9000 *g.*
11. Aspirate the supernatant (here LB).

***Note:****Salmonella* pellets can easily detach within a few min. Act fast and leave a safe volume above the pellet to avoid discarding bacteria.

12. Wash the bacteria with 1 mL of pre-warmed DMEM-.
13. Spin down for 1 min at 9000 *g.*
14. Aspirate the supernatant (here DMEM-).
15. Resuspend the bacterial pellet in 1 mL of pre-warmed DMEM-.
16. Prepare the infection mix by diluting the bacteria in DMEM- according to the OD600 measured and the MOI wanted. Here is a detailed example of calculations:
  a. We expect a cell confluency of circa 90%, corresponding to 2 × 10^5^ cells per well. Troubleshooting 4. For an MOI of 20, 4 × 10^6^ bacteria should be added per well.
  b. The ideal volume of infection mix to add per well is 0.5 mL. This allows covering the whole well while using a minimal volume to facilitate bacteria-cell interactions. To add 4 × 10^6^ bacteria to the cells within a volume of 0.5 mL, the bacteria concentration should be 2 × 4 × 10^6^ = 8 × 10^6^ bacteria/mL.
  c. To infect 2 wells, prepare enough volume for the number of wells + 0.150 mL to account for loss during pipetting. Here the volume is 2 × 0.5 mL + 0.150 mL extra = 1.150 mL.
  d. For an OD600 of 2 and assuming a conversion factor OD600 to CFU of 6.5 × 10^8^, the concentration of bacteria in the subculture is 2 × 6.5 × 10^8^ = 1.3 × 10^9^ bacteria/mL.
  e. Thus, 1.150 mL of “infection mix” can be prepared by diluting 7 μL of washed bacterial subculture into 1.143 mL of DMEM-.

**CRITICAL:** The conversion factor from OD600 to CFU to use should be established in each lab during spectrophotometer calibration.

17. Take the 12-well plate containing the cells from the incubator.
18. Remove the cell medium and add 0.5 mL of infection mix to the cells of the “infected” conditions and 0.5 mL DMEM- to the cells of the “non-infected” condition.
19. Put the cells back in the incubator at 37°C, 5% CO_2_ for 30 min.
20. Wash the cells twice with pre-warmed DMEM-.
21. Add 1 mL of pre-warmed DMEM+FBS+Gent100 per well.
22. Put the cells back in the incubator at 37°C, 5% CO_2_ for 1 h.

***Note:*** The washes remove most of the extracellular bacteria. The remaining extracellular bacteria attached to the cells will be killed by gentamicin. As gentamicin is not cell permeable, the intracellular bacteria are not affected by the gentamicin treatment.

***Optional:*** In case the *S*. Typhimurium strain expresses a fluorophore, the efficiency of bacteria infection can be observed on a (bench-top) fluorescence microscope, if available.

23. Aspirate the medium and replace it with 1 mL pre-warmed DMEM+FBS+Gent10.

***Note:*** The concentration of gentamicin is decreased after 1 h to avoid the accumulation of intracellular gentamicin (especially by endocytosis) that could affect intracellular bacteria.

24. Put the cells back in the incubator at 37°C, 5% CO_2_ for 2 h.

### Agar top layer

**Timing: 30 min, followed by 2–3 weeks of incubation**

Prepare the 0.45% agar top layer containing the cells.

**CRITICAL:** Make sure that all media are at 37°C and remelt the 3% soft agar solution if it is no longer fully liquid (Figures 1A-1C). Keep the 3% soft agar solution at 40°C–42°C using a water bath or a ThermoMixer with 300 rpm agitation (Figure 1D).

25. Collect the cells from the 12-well plate:
  a. Remove the medium from each well.
  b. Gently wash each well with 1 mL of PBS.
  c. Remove PBS.
  d. Add 200 μL of trypsin per well.
  e. Incubate the cells for 5 min at 37°C.
  f. Verify that cells are ready to detach using a bench microscope.
  g. Add 800 μL of DMEM+FBS per well to stop the action of trypsin.
  h. Resuspend cells and pull 2 wells corresponding to 1 condition in one 15 mL centrifuge tube (so in total 2 mL per tube).
  i. Spin down at 300 *g* for 4 min.
  j. Remove the supernatant and resuspend every pellet in 1 mL of DMEM+FBS+Gent10.
26. Count the cells for each condition.

***Note:*** Various cell counting methods exist. Cells can be counted manually or automatically using counting chambers or automated cell counters, respectively. While automated cell counting is less time-consuming, manual counting is less expensive.^5^ Both types of cell counting methods can be used for this step.

27. For each condition, transfer 2 × 10^5^ cells in a new 15 mL centrifuge tube and complete the volume to 6.8 mL with DMEM+FBS+Gent10. Troubleshooting 5.
28. Add 1.2 mL of melted 3% soft agar to one of the cell-containing centrifuge tubes resulting in a final agar concentration of 0.45% and a final volume of 8 mL.

**CRITICAL:** Perform Steps 28–32 fully for one condition before moving to the next. Adding soft agar to all samples at once would risk agar solidification before pouring.

**CRITICAL:** The 3% soft agar solution should be fully melted but not too hot to avoid damaging the cells. Ideally, the solution should be maintained around 40°C–42°C using a water bath or a ThermoMixer.

29. Using a 5 mL pipette, take 2.5 mL of the cell and agar-containing solution (Methods video S1).

30. Pass the cells through a 5 mL polystyrene round-bottom tube with cell-strainer cap (Methods video S1).
31. Collect the solution in the round-bottom tube and pass it through a second cell strainer cap. This will avoid cell clumping. Use the pipetboy gently to avoid bubbles (Methods video S1).
32. Pour 2 mL of the solution on the top of the agar bottom layer. Thus 5 × 10^4^ cells are seeded per well (Methods video S1). Troubleshooting 6–7.
33. Repeat Steps 29–32 twice more starting from the same cell and agar-containing tube to fill 3 wells of the 6-well plate per condition.

**CRITICAL:** The addition of melted soft-agar to the cell suspension and the pouring of the solution are technically challenging steps that can introduce important variations to the results. Thus, it is recommended to use technical triplicates.

34. Repeat Steps 28–33 with the next condition.
35. Carefully transfer the 6-well plate to a flat shelf of a fridge at 4°C.
36. Let the plate cool down for 20 min until the top layer is fully solidified. Troubleshooting 8.
37. Fill the spaces between the wells with sterile water or PBS to prevent the fast drying of the agar gel.
38. Incubate at 37°C for 2–3 weeks (or until cell colonies are visible). Troubleshooting 9–10.

Methods Video S1. Filtering and deposition of the top agar layer, related to Steps 29–32

### Image acquisition of the plate

**Timing: 1 h**

When small cell colonies are visible, acquire the 6-well plate using the GelCount device and the GelCount Software.

***Note:*** In case a GelCount device is not available, manual counting of the colonies using a bench microscope can be performed. Of note, manual counting is more time-consuming, labor-intensive, and subjective than automated detection.

**CRITICAL:** cell colonies should be counted before they grow too much and overlap. Check the plate regularly with a cell culture microscope to see if cell colonies start forming (several cells attached together are considered as a forming colony).

**Figure 2:**
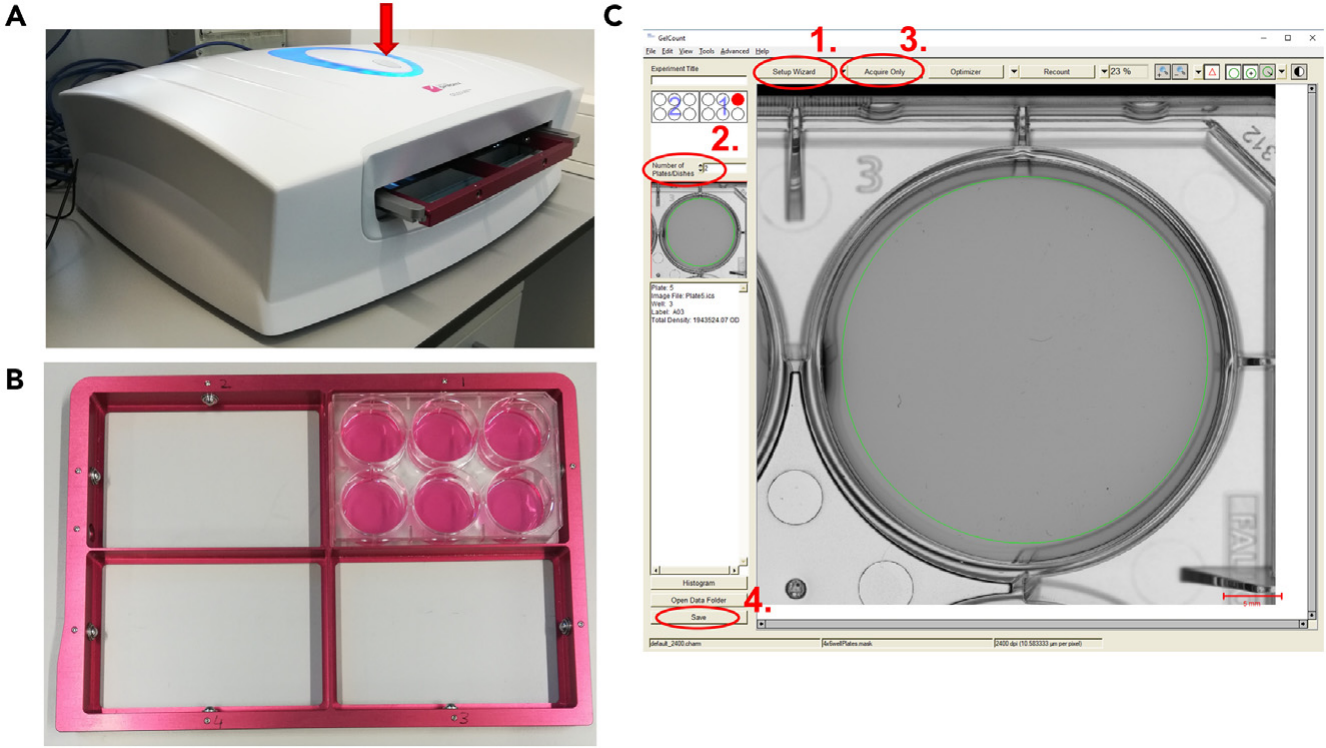
Plate acquisition with the GelCounter device (A) GelCounter device, red arrow: insert button. (B) 6-well plate tray. (C) Screenshot of the GelCounter software used for image acquisition. Related to Steps 39–49.

39. Turn on the GelCount device using the back on/off button (Figure 2A). The plate tray is then ejected from the device (Methods video S2).

40. Extract the 6-well plate tray (Figure 2B) and place it under the hood.
41. On the computer connected to the GelCount device, start the GelCount software.
42. Click the “Setup wizard” button (Figure 2C, circle 1), and select a 6-well plate format and a resolution of 2400 dpi. Troubleshooting 11.
43. Wipe the bottom of the 6-well plate(s) with 70% ethanol to remove dust. Troubleshooting 12.
44. Place the 6-well plate(s) within the tray and take a picture of the plate positions (Figure 2B).
45. Take off the cap of the 6-well plate(s).

***Note:*** The acquisition of the colony on the GelCount device will likely compromise the sterility of the culture. However, if the plate needs to be acquired several times, a broad-spectrum antibiotic such as Primocin at 100 μg/mL can be spread over the agar top layer to prevent microbial contamination.

46. Insert the tray with the plate within the GelCount device and press the central button to close the device (Figure 2A, red arrow).
47. On the GelCount software, select the number of plates to acquire (Figure 2C, circle 2).
48. Press “acquire only” (Figure 2C, circle 3). The acquisition will take around 5–10 min per plate.
49. Save the files (Figure 2C, circle 4).

***Note:*** The quantitative analysis of the cell colonies can be done at a later time on any off-line computer equipped with the GelCount software (i.e., it is not required to use the computer connected to the GelCount device).

Methods Video S2. Image acquisition of the plates, related to Steps 39–48

## Expected outcomes

**Figure 3:**
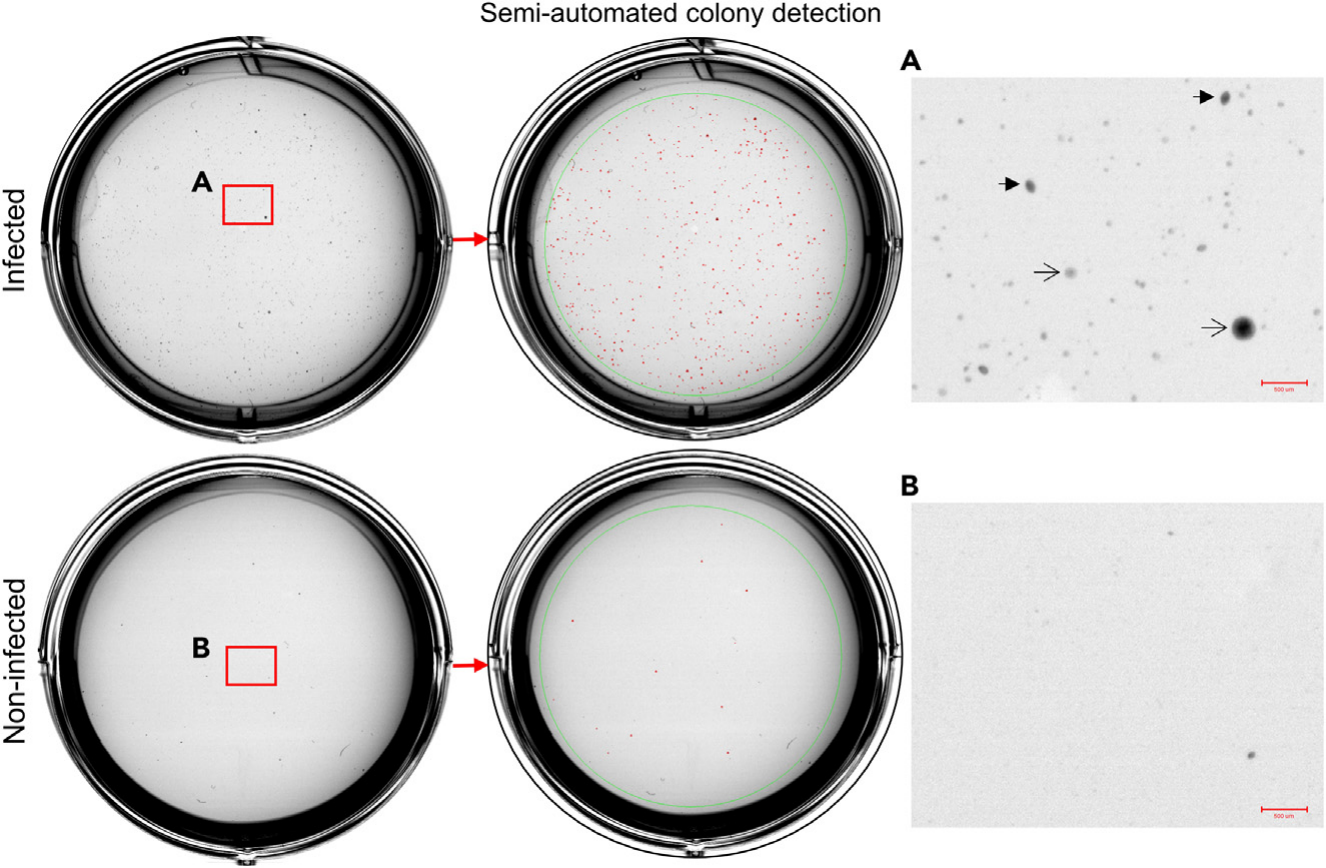
Expected outcome after 2–3 weeks of growth Cell colonies are visible to the naked eye in the 6-well plate. The infected condition contains >100 cell colonies while the non-infected condition contains only a few colonies. Red dots: cell colonies automatically detected. Open arrows: round shape cell colonies. Full arrows: almond shape colonies. Red scale bar: 500 μm. Related to Expected outcomes.

The expected outcome is the observation of ≈100–1000 cell colonies in the “infected” condition and none to very few in the “non-infected” condition (Figure 3). Cell colonies should be larger than 80 μm diameter and may have round or almond shapes (Figure 3). Besides, the number of cell colonies should be similar between experimental triplicates.

## Quantification and statistical analysis

***Note:*** The automatic detection of cell colonies described below uses the GelCount software. However, open-source Fiji plugins can fulfill the same purpose.

**Figure 4:**
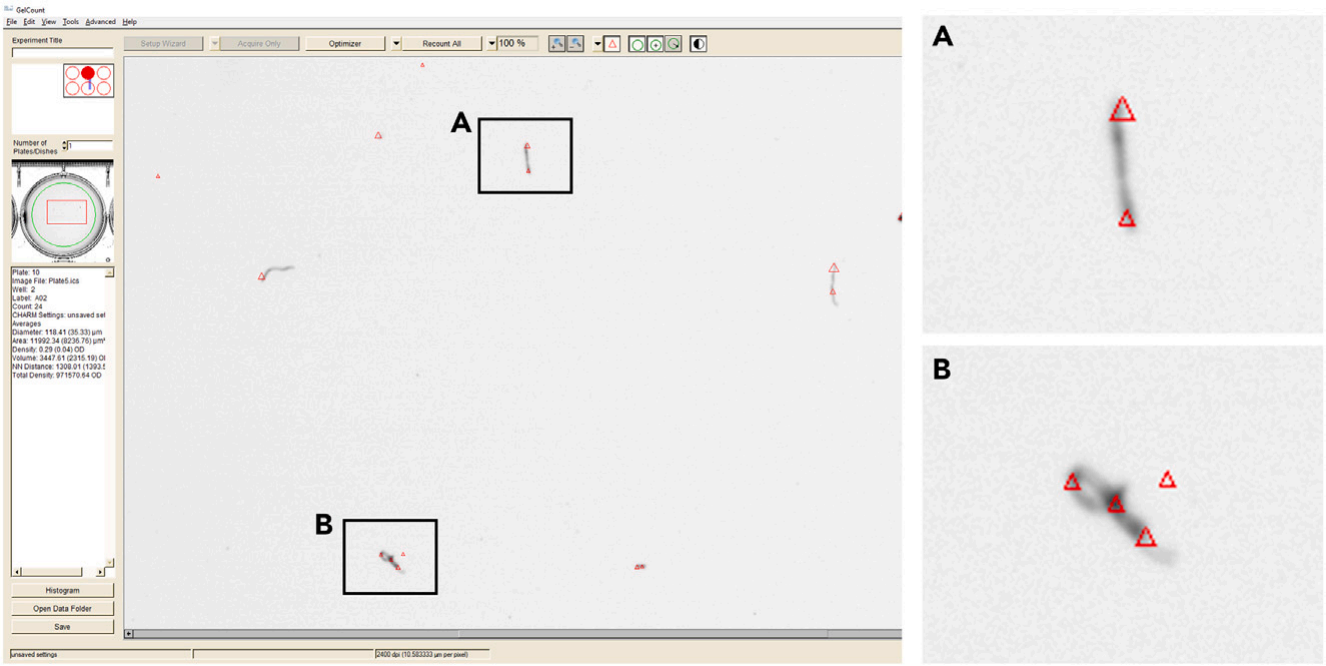
Dust detected as cell colonies that must be removed manually, related to Quantification and statistical analysis

***Note:*** Ideally, 3 wells should be counted per condition. The data can be visualized on graphs using Excel or Prism. Statistical analyses such as unpaired t-tests can be performed to test significant differences between the tested conditions.

1. Open the GelCount software (Methods video S3).

2. Press “File” and select “Open Multiple Plates/Dishes” in the menu (Methods video S3).
3. Select the data that was saved after the plate acquisition. Press “Add to” to see the plate acquisition file appearing in the right window and press Ok. If several plates were acquired, add all corresponding files (Methods video S3).
4. Select the size and position of the well mask using the green circles in the top bar. The selected area should not include the gel border or its shadow (Methods video S3).
5. Press “Tools” and select “Apply this Mask to all”. If necessary, reposition the mask in other wells but keep the area identical (Methods video S3).
6. Press “Optimizer” to open the automatic colony detection tool (Methods video S3).
7. Adjust the settings depending on the size and density of the cell colonies. Press “Ok”.
8. Press the arrow next to the “Recount” button and select “Recount all”. Thus, all wells will be counted using the same detection settings. The number of cell colonies counted appears in the left column (Methods video S3).
9. To manually deselect some false positive cell colonies such as dust, maintain the “Ctrl” key pressed and right-click on the detected colony to delete it (Figure 4).

Methods Video S3. Automated detection and counting of the colonies, related to Quantification and statistical analysis

## Limitations

The protocol described may provide unreliable results if the cell line used is spontaneously producing cell colonies in soft agar (independently of infection^6^^,^^7^^,^^8^^,^^9^) or if the cell viability is compromised before or during soft agar growth. In these cases, the number of cell colonies detected may be independent of the infection state of the cells. Environmental factors when handling the cells may impact their ability to form cell colonies such as starvation, too high confluency, high passage number, etc. Besides, while additional treatment of the cells, for instance with drugs, may be used to study the mechanism of bacterial-induced cell transformation, cell viability assays must be performed to select a drug concentration that does not impact cell viability upon long exposure. Troubleshooting 13.

The infection efficiency is another factor that may impact the number of transformed cells. This parameter should be evaluated independently, in particular when comparing the transformative impact of different bacterial strains. It is possible to change the MOI to increase or decrease the percentage of infected cells (using the calculation provided in Step 16).

The soft agar cell transformation assay allows quantifying cell transformation in an unbiased manner (i.e., without focusing on a specific pathway) in a cost-effective and simple *in cellulo* system. However, more complex systems (such as organoids or *in vivo* models) can be used to measure bacteria-induced transformation.^2^

## Troubleshooting

### Problem 1

The subculture does not reach an OD600 of 2 in 3 h of growth (related to Steps 1 and 8).

### Potential solution

• An OD600 of 2 is usually reached in 3 h but this might vary between labs.
• The time of culture to reach an OD600 of 2 can be previously tested by measuring the OD600 of the subculture at regular intervals.
• In case of punctual variation of growth speed, use fresh LB medium, and freshly streaked bacteria colonies.
• Make sure that the subculture has access to air and that the incubator with orbital shaker has a stable temperature of 37°C.

### Problem 2

Air bubbles are created (related to Step 4).

### Potential solution

• Air bubbles compromise the homogeneity of the bottom layer and could lead to cells reaching the bottom of the plate.
• If formed, air bubbles can be removed with a pipet before soft agar solidification by carefully aspirating the air bubble without removing the liquid soft agar.
• If air bubbles can not be removed, trash this bottom layer and make a new one.

### Problem 3

The bottom layer is not completely solidified after 20 min at 4°C (related to Step 6).

### Potential solution

• This is an indication that something went wrong with the preparation of the bottom layer.
• Trash this bottom layer and make a new one. Going any further with a suboptimal agar gel is likely to lead to experimental failure.
• If the same problem happens again, this suggests that the bottom layer is too fluid. Then, make a new 3% agar solution.

### Problem 4

The cells are too dense or too sparse on the infection day (related to Step 16).

### Potential solution

The cell speed of growth can vary between labs and over culturing time (i.e., number of passages). If cells are stressed due to too high confluency, this may compromise the reproducibility of the results. Besides, the cell density will influence the probability of successful bacterial infections.^10^ Adjust the plating concentration accordingly (“before you begin”). Optionally, plate different concentrations as a backup.

### Problem 5

There are less than 2 × 10^5^ cells recovered after trypsinization (related to Step 27).

### Potential solution

• If the low cell density was observed before infection, see problem 3.
• If the cells displayed the appropriate confluency but not enough cells are measured in Step 27, this suggests that either the cells detached during the infection and washing steps, or that the trypsinization step was not efficient enough to collect all cells.
• Prevent cell detachment during the infection and washing steps by gently adding and removing the medium from the well.
• Make sure that all cells are collected after the trypsinization step. Check at the microscope if some cells remained attached. Pipette the medium several times on the well while keeping the plate tilted to ensure collecting a maximum of cells. Increase trypsinization time if necessary.

### Problem 6

The bottom layer detaches when you pour the top layer (related to Step 32).

### Potential solution

This suggests that the bottom layer is not concentrated enough or did not cool down properly. See problem 2. It is possible to use different agar concentrations: for the bottom layer, from 0.5% to 1%; for the top layer, from 0.25% to 0.5%. Use a bottom layer that is twice more concentrated than the top layer.

### Problem 7

The layers of agar are heterogeneous (related to Step 32).

### Potential solution

Make sure the agar is melted without clumps before using it. If your 3% agar solution starts solidifying (appearance of clogs and viscosity), heat it again and let it cool down to 40°C–42°C before proceeding to dilution. Use a water bath or a ThermoMixer to prevent temperature variation. If none of these are available, put the bottle with agar in a beaker with hot water.

### Problem 8

The top layer is not completely solidified after 20 min at 4°C (related to Step 36).

### Potential solution

This is an indication that something went wrong with the preparation of the top layer. The risk is that the cells will sediment. It is recommended to repeat the preparation of the top layer.

### Problem 9

The well dries up before the cell colonies are big enough to be observed (related to Step 38).

### Potential solution

• The PBS added between wells should prevent the drying of the soft agar. If the PBS is fully evaporated, add new PBS.
• Make sure the incubator contains water to maintain humidity. 90% humidity is ideal to limit evaporation from culture plates. To create humidified atmosphere, a large water tank should be placed at the bottom of the incubator.^11^
• A layer of growth medium can also be added to the top layer of agar to prevent desiccation. Another protocol recommends adding 100 μL of medium twice weekly.^8^

### Problem 10

Cell colonies are hard to detect due to a high background or small size of the cell colonies (related to Step 38).

### Potential solution

• Incubate the plate longer to let the cell colonies grow.
• Use cell viability staining such as MTT-based metabolic stains that do not stain the medium to increase the contrast between colony and background.
• It is possible that the cells did not survive the embedding in agar, for instance, if the agar was too hot. To test this, include a positive control such as carcinoma cell lines that spontaneously grow in soft agar.^6^^,^^7^^,^^8^^,^^9^

### Problem 11

Artifacts appear on the image acquired on GelCount acquisition (related to Step 42).

### Potential solution

Others have previously reported artifact appearance when using 2400 dpi and suggest using 1200 dpi during acquisition.^7^

### Problem 12

Dust particles are detected as cell colonies on GelCount (related to Step 43).

### Potential solution

• Make sure to wipe the bottom of the plate with 70% ethanol before acquisition. This will prevent the electrostatic attraction of dust particles.
• Deselect manually the dust on the GelCount software (Figure 4).

### Problem 13

The experimental design requires the use of unstable molecules to grow the cells (inhibitor, growth factor, etc).

### Potential solution

• Add medium supplemented with the necessary molecules on top of the top layer.
• Every other day, remove the deprived medium and replenish the gel with a fresh medium.
• Be careful not to disturb the gel when removing the deprived medium.

## Resource availability

### Lead contact

Further information and requests for resources and reagents should be directed to and will be fulfilled by the lead contact, Virginie Stévenin, virginie.stevenin@ens-cachan.fr.

### Materials availability

This study did not generate new unique reagents.

## Data Availability

This study did not generate code. The published article includes all datasets analyzed during this study.
